# Advances in Nanotoxicology: Towards Enhanced Environmental and Physiological Relevance and Molecular Mechanisms

**DOI:** 10.3390/nano11040919

**Published:** 2021-04-04

**Authors:** Anne Kahru, Monika Mortimer

**Affiliations:** 1 Laboratory of Environmental Toxicology, National Institute of Chemical Physics and Biophysics (NICPB), Akadeemia Tee 23, 12618 Tallinn, Estonia; 2 Institute of Environmental and Health Sciences, College of Quality and Safety Engineering, China Jiliang University, Hangzhou 310018, China

Nanotoxicology, a discipline transpired by the need to assess the human and environmental safety of nanoscale materials, has evolved over the past 15 years into a mature area of toxicology. Early on, nanotoxicology studies established a necessary understanding of dose-response relationships of nanomaterials using typical in vitro toxicology models, provided crucial information on interferences induced by nanoparticles (NPs) in the conventional toxicity assays and how to overcome these, and demonstrated that due to their unique physical–chemical properties, nanomaterials act via different toxicological mechanisms than the bulk counterparts of the same materials. For example, ZnO, known as a water-insoluble compound in bulk form, was shown to exert toxicity through released Zn-ions in the case of ZnO NPs, and the cellular uptake pathways of NPs were discovered to depend on the size, shape, and surface properties of nanoscale particles. As the knowledge on NP toxicity mechanisms and behavior in the environment and organisms has accumulated, nanotoxicologists have acknowledged the need for improved understanding about the NP interactions with biological systems at the molecular level to better predict the potential toxicity of novel nanomaterials and ensure safe and sustainable development of nanotechnology.

Accordingly, recent trends in nanotoxicology include incorporation of high-throughput screening approaches to establish structure-activity relationships using libraries of NPs with different properties and in vitro screening assays combined with computational analysis; omics- approaches, including metabolomics, proteomics, and transcriptomics, to discover molecular-level effects of NPs at low, sub-lethal NP concentrations, and method development to include sensitive toxicity assays for early detection of toxic effects. Environmental nanotoxicology is moving towards the direction of enhancing the relevance of testing conditions (e.g., media composition, exposure concentrations, and duration) to these relevant to the environment and organism physiology, because NP toxicity has been proven to depend on the interactions with natural organic matter, anions and cations, the level of pH, and other environmental factors. Another major direction of nanotoxicology which has gained momentum in the recent years is the safety assessment of nanomaterials designed for applications in biotechnology, environmental bioremediation, wastewater treatment, agriculture, and nanomedicine. Thus, nanotoxicology has a substantial role not only in the risk assessment of unintentionally or intentionally produced nanomaterials but also in ensuring successful nanoinnovation across broad applications and enabling safe-by-design approach in nanotechnology.

This Special Issue covers a range of topics regarding the latest research trends in nanotoxicology. The collection includes 10 research articles and 5 reviews by authors from 16 countries, spanning four continents, which illustrates the global significance of nano-enabled technology and its safety assessment. It is worth noting that, in addition to guest editors who both are female scientists, most of the lead authors of the papers of this Special Issue are female scientist which is an important tendency in the STEM areas. The topics of the research articles and review papers address both environmental nanotoxicity as well as human health and nanomedicine safety aspects, providing an excellent overview of the current status of these important sub-areas of nanotoxicology.

The original research articles regarding environmental nanotoxicology address aspects that have been less explored in the nanotoxicology studies so far, such as combination toxicity and long-term toxicity of NPs. For example, Wang et al. investigated the toxicity of the binary mixture of TiO_2_ nanospheres and TiO_2_ nanotubes to two freshwater algae with different morphology [1]. They found that the combined toxicity of TiO_2_ nano-spheres and nanotubes was different from the toxicity of single exposures and depended on the microalgal species. Aravantinou et al. assessed the long-term toxicity of ZnO NPs to microalgae using a modeled natural water treatment system with a semi-continuous supply of NPs [2]. A freshwater microalgal species *Scenedesmus rubescens* was used as a model due to its presence in municipal wastewater and its potential use for biofuel production. The study showed that low concentration (0.081 mg/L) of ZnO NPs, supplied at lower hydraulic retention time, slightly inhibited algal growth but enhanced the lipid content of *S. rubescens*, highlighting the need for further studies of NP-biota interactions in environmentally relevant conditions. The importance of considering the presence of natural organic matter in nanotoxicity studies has been well illustrated in the article by Bondarenko et al. who assessed the ecotoxicity of magnetite NPs in a plant and unicellular eukaryotic model, assessing the influence of humic acid on the toxicity of the NPs as well as Fe(II) and Fe(III) ions [3]. In another research article, Bondarenko et al. assessed the biological effects of magnetite NPs on bacteria and their enzymatic reactions and the role of humic substances and silica in biogeochemical cycling of iron. The article also proposes whole-cell and enzyme-based bioluminescence assays as convenient tools to evaluate bio-availability of Fe(III) in natural dispersions of iron-containing NPs [4]. Development of novel cell-based toxicity assays was also the focus of the article by Semerad et al. who addressed the issue of potential toxicity of NPs applied for remediation in soils and groundwater, to soil organisms [5]. They used amoebocytes, the immune effector cells of the earthworm *Eisenia andrei*, to study the toxicity mechanisms of nanoscale zero-valent iron (nZVI).

The method development and application of novel omics approaches, which can provide molecular-level information about NP biological effects, were also the focus of research articles regarding the human health and nanomedicine safety. Enea et al. applied an innovative and sensitive approach—metabolomics—to compare the in vivo toxicological effects of gold nanospheres versus gold nanostars of similar ~40 nm diameter, coated with 11-mercaptoundecanoic acid, 24 h after an intravenous administration to Wistar rats [6]. Using a metabolomic approach, different results were obtained with gold nanospheres and gold nanostars in the liver. Subtle changes were found at the molecular level that preceded the conventional toxicity symptoms and biochemical changes, allowing the discrimination between the effects of different Au NPs at subtoxic doses. In the future studies of Au NPs the authors recommend focusing on fatty acid synthesis and metabolism of pyrimidine and purine. In another research article, Tomonaga et al. propose cytokine-induced neutrophil chemoattractants CINC-1 and CINC-2 as early biomarkers for the prediction of pulmonary toxicity of NPs [7]. Nguyen et al., on the other hand, compared the performance of monolayer cell cultures versus multicellular tumor spheroids in estimating the highest tolerable dose of cancer medicines delivered by NPs [8]. They used superpara-magnetic iron oxide nanoparticles (SPIONs) functionalized with mitoxantrone (MTO) for targeted delivery of the chemotherapeutic and demonstrated that multicellular tumor spheroids tolerated higher doses of NP-loaded MTO than monolayer cell culture. The study highlights the issues of using conventional monolayer cell cultures for drug dose predictions which may be inaccurate for in vivo applications. Geng and colleagues explored the mechanisms by which ultraviolet A (UVA) irradiation exacerbates TiO_2_ NP toxicity, and specifically clarified the mechanism of cell death caused by the combined exposure to UVA and TiO_2_ NPs [9]. They investigated the cytotoxicity and phototoxicity of mixture crystalline TiO_2_ NPs (25% rutile and 75% anatase, 21 nm) under UVA irradiation in HeLa cells and showed that the abnormal membrane integrity and the ultrastructure of HeLa cells, together with the decreased viability induced by TiO_2_ NPs under UVA irradiation, were due to cell necrosis rather than caspase-dependent apoptosis.

Several articles in the Special Issue discuss the beneficial properties on NPs and the associated mechanisms as well as safe environmental application of NPs. In their research paper, Sguizzato et al. report on the development of a formulation for caffeic acid cutaneous administration [10]. The nanoformulation has the benefits of easy cutaneous application and improved properties to combat inflammation, microbial infections, and carcinogenic effects. The review by Zhou et al. provides an overview and a synthesis of the published literature regarding the NP applications for plant stress alleviation and growth stimulation [11]. By discussing the mechanisms of interaction between NPs and heavy metals, the review provides valuable information for NP applications in phyto- and soil remediation as well as sustainable agriculture. Malhotra et al. review published data regarding the toxicity of Cu and Cu NPs in various fish species [12]. Since Cu-based formulations are widely applied as antifouling paints in underwater surfaces, understanding the toxicity and mechanisms of action of Cu NPs are crucial for establishing guidelines for the usage of these NPs in a sustainable manner.

Clinical applications of NPs and associated safety concerns have been discussed in three review articles. Mosselhy et al. review recent advances in nanotheranostics focusing on the issue of detecting methicillin-resistant *Staphylococcus aureus* (MRSA) and biofilm eradication in clinical settings [13]. The authors propose three action levels to quickly detect and combat the concerning issue of MRSA. The review by Damasco et al. discusses preclinical and clinical inorganic NPs utilized for cancer imaging and therapeutics [14]. A special emphasis is put on the rational design to develop non-toxic/safe inorganic NP constructs to increase their viability as translatable nanomedicine for cancer therapies. Lama et al. analyze the potential oral toxicity mechanisms of nanoformulations and describe recently reported in vitro and in vivo models that have been used to evaluate the oral toxicity of nanomedicines [15]. The authors also discuss approaches that may be used to develop nontoxic nanoformulations for oral drug delivery.

In summary, the article collection in this Special Issue addresses important recent trends in nanotoxicology, both from the environmental and human health safety aspects. The articles illustrate the directions where the toxicity studies of NPs (including various nanoformulations designed for applications in agriculture, bioremediation, and nanomedicine) are headed: employing novel and sensitive methods which provide molecular level information about the mechanisms of action of NPs, focusing on the NP-biomolecule interactions, development of biomarkers for early detection of NP toxicity, and guiding safe-by-design strategies for NPs.
